# Renal Function and Death in Older Women: Which eGFR Formula Should We Use?

**DOI:** 10.1155/2017/8216878

**Published:** 2017-03-29

**Authors:** Muna T. Canales, Terri Blackwell, Areef Ishani, Brent C. Taylor, Allyson Hart, Rebecca J. Beyth, Kristine E. Ensrud

**Affiliations:** ^1^Malcom-Randall VAMC, Department of Medicine, University of Florida, Gainesville, FL, USA; ^2^Research Institute, California Pacific Medical Center, San Francisco, CA, USA; ^3^Department of Medicine, University of Minnesota, Minneapolis VA Health Care System, Minneapolis, MN, USA; ^4^Center for Chronic Disease Outcomes Research, Minneapolis VA Health Care System, Department of Medicine, University of Minnesota, Minneapolis, MN, USA; ^5^Hennepin County Medical Center, Minneapolis, MN, USA; ^6^Malcom-Randall VAMC GRECC, Department of Medicine, University of Florida, Gainesville, FL, USA

## Abstract

*Background*. The Berlin Initiative Study (BIS) eGFR equations were developed specifically for aged populations, but their predictive validity compared to standard formulae is unknown in older women.* Methods*. In a prospective study of 1289 community-dwelling older women (mean age 79.5 years), we compared the performance of the BIS1 SCr-based equation to the CKD-EPI_cr_ and the BIS2 SCr- and Scysc-based equation to the CKD-EPI_cr,cysc_ to predict cardiovascular and all-cause mortality.* Results*. Prevalence of specific eGFR category (i.e., ≥75, 60–74, 45–59, and <45) according to eGFR equation was 12.3%, 38.4%, 37.3%, and 12.0% for BIS1; 48.3%, 27.8%, 16.2%, and 7.8% for CKD-EPI_cr_; 14.1%, 38.6%, 37.6%, and 9.6% for BIS2; and 33.5%, 33.4%, 22.0%, and 11.1% for CKD-EPI_cr,cysc_, respectively. Over 9 ± 4 years, 667 (51.8%) women died. For each equation, women with eGFR <45 were at increased risk of mortality compared to eGFR ≥75 [adjusted HR (95% CI): BIS1, 1.5 (1.1–2.0); CKD-EPI_cr_, 1.7 (1.3–2.2); BIS2, 2.0 (1.4–2.8); CKD-EPI_cr,cysc_, 1.8 (1.4–2.3);* p*-trend <0.01]. Net reclassification analyses found no material difference in discriminant ability between the BIS and CKD-EPI equations. Results were similar for cardiovascular death.* Conclusions*. Compared to CKD-EPI, BIS equations identified a greater proportion of older women as having CKD but performed similarly to predict mortality risk. Thus, the BIS equations should not replace CKD-EPI equations to predict risk of death in older women.

## 1. Introduction

Reduced renal function defined by varying cut-points of estimated glomerular filtration rate (eGFR) in older adults has been associated with greater risk of cardiovascular (CVD) and all-cause mortality [1–3]. However, there is debate regarding the threshold of eGFR beyond which risk of death rises [4, 5]. Much of this controversy is related to the insensitivity of serum creatinine (SCr) based estimates in older adults and the lack of eGFR formulae developed specifically for use in the aged population [6].

In an effort to move toward more accurate estimation of GFR across populations and age groups, the 2012 Kidney Disease Improving Global Outcomes (KDIGO) guidelines called for a paradigm shift from use of the clinical standard Modification of Diet in Renal Disease (MDRD) formula to the Chronic Kidney Disease Epidemiology Collaboration creatinine (CKD-EPI_cr_) 2009 equation to estimate GFR in adults [7]. Furthermore, for the aged population in which SCr-based estimates may not be as sensitive, the guidelines recommend use of serum cystatin C (Scysc) based equations (CKD-EPI_cysc_ and CKD-EPI_cr,cysc_) [7]. The CKD-EPI formulae were developed in a large population (*n* = 8254 and 5352 for the CKD-EPI_cr_ and the CKD-EPI cystatin C equations, resp.) that pooled participants from various research studies and clinical cohorts. While the CKD-EPI cohorts reflected gender and racial/ethnic diversity (56–58% male, 30–40% African-American) and a range of renal function (mean measured GFR 68 (±40) mL/min/1.73 m^2^), they underrepresented older adults (mean age was 47 ± 15 years) [6, 8]. Thus, KDIGO guidelines further recommended the use of alternate formulae that may perform better for certain populations with respect to estimation of GFR [7].

To that end, the Berlin Initiative Study 1 (BIS1, SCr-based) and BIS2 (SCr and Scysc-based) were the first eGFR formulae to be developed and internally validated in comparison to direct measures of GFR specifically in a population of older adults using a cohort of 570 community-dwelling older Caucasian German men and women over 70 years of age with mean measured GFR (range) 60 (16–117) mL/min/1.73 m^2^ [9]. These formulae represent the first attempt to tailor a formula to the aged population while also incorporating a more sensitive measure of renal function in the elderly, Scysc [9]. To date, however, no study has validated the performance of these equations against the KDIGO-recommended CKD-EPI equations to predict the key outcome of death in older women.

Therefore, we studied 1289 participants enrolled in a multicenter, prospective study of disease outcomes in community-dwelling older women, the Study of Osteoporotic Fractures (SOF), who had existing measures of both SCr and Scysc, and compared the ability of the new BIS1 (SCr-based) and BIS2 (SCr and Scysc-based) equations to guideline-recommended CKD-EPI_cr_ and CKD-EPI_cr,cysc_ equations, respectively, to predict incident all-cause and CVD death.

## 2. Methods

### 2.1. Participants

The SOF is a NIH-funded prospective cohort study of older women initially designed to determine risk factors for fractures and falls but expanded to evaluate risk for other outcomes including all-cause and cause-specific mortality in older women. SOF enrolled 9704 women aged ≥65 years between September 1986 and October 1988 for participation in the baseline examination [10]. Participants were recruited from population-based listing from four regions in the United States (Baltimore, MD, Pittsburgh, PA, Minneapolis, MN, and Portland, OR) [10]. Women with a history of bilateral hip replacement or who were unable to walk without the assistance of another person were excluded from participation. Black women were also excluded in the original enrollment for SOF given a low incidence of hip fracture in this demographic. Subsequently, at the Year 10 SOF examination conducted between 1997 and 1998, 662 African-American women were enrolled in the study increasing the total cohort enrollment to 10,366 women [11].

Of the original 9704 white women, 7008 (96% of active participants) completed at least the questionnaire component of a Year 10 examination (between 1997 and 1998). Of those 2696 women who did not participate, 2081 had died before the visit and 290 had terminated study participation. All 662 African-American women completed this examination, bringing the total enrollment at the Year 10 examination to 7670. Of the 7670, 5470 completed an in-clinic examination of which 5346 provided serum specimens. Of the 5346, a subset of 1302 was randomly selected for renal function measurement. Of these, 1289 women with measurements of both SCr and Scysc at the time of the Year 10 exam and data available regarding vital status through April 2012 comprised the analytical cohort for this study. Thus, the observation period for this study spans the time of the Year 10 exam (1997-1998) with follow-up for the primary outcome of death through April 2012 (mean follow-up for mortality 9 ± 4 years).

All women provided written informed consent, and the study was approved by the Institutional Review Board at each site.

### 2.2. Estimation of Renal Function

Serum from fasting morning blood collected at the Year 10 examination was processed and stored at −70°C until being thawed. SCr and Scysc levels were measured on these previously frozen serum samples. Scysc and SCr assays were performed at the University of Minnesota Medical Center in 2010. Scysc concentrations were determined using a BN100 nephelometer (Dade Behring Inc., Deerfield, IL) using a particle-enhanced immunonephelometric assay (assay range 0.23–8.00 mg/L with interassay coefficient of variation (CV) of 4.0% at a level of 0.71 mg/L and 3.1% at a level of 1.75 mg/L [mean interassay CV 3.7%]) and then converted to standardized values traceable to a certified reference material. SCr was measured with a Modular P Chemistry Analyzer (Roche Diagnostics, Indianapolis IN) using an enzymatic method equation calibrated with materials traceable to an isotope-dilution mass spectrometry (IDMS) reference measurement procedure. Interassay CV is 4.0%.

We estimated GFR using the BIS1, CKD-EPI_cr_, BIS2, and CKD-EPI_cr,cysc_, and equations are shown in Appendix Table 1 in Supplementary Material available online at https://doi.org/10.1155/2017/8216878.

### 2.3. Mortality

Women were contacted every 4 months after the Year 10 examination until May 2012 to ascertain vital status (mean follow-up 9 ± 4 years); 95% of these contacts were completed in surviving participants. Cause of death was confirmed by death certificate and, when available, hospital discharge summaries. We used ICD-9 codes to classify causes of death as CVD (codes 401–405, 410–415, 425, 427.5, 428, 429.2, 430–439, 440–445, and 798).

### 2.4. Other Measurements

Demographic data such as race and education level were taken from baseline examination for white women and Year 10 examination for Black women. Body-mass index (BMI, kg/m^2^) as a measure of obesity, smoking status, and self-reported health status were obtained at the Year 10 examination. Comorbid conditions as defined by self-report alone at the Year 10 examination included stroke, coronary heart disease (CHD), and congestive heart failure (CHF). Hypertension (HTN) and diabetes mellitus (DM) were defined as self-report or use of antihypertensive or antihyperglycemic medications, respectively.

### 2.5. Statistical Analysis

We used the Chi-square test to compare the prevalence of CKD in 4 categories of eGFR (>75, 60–74, 45–59, and <45 mL/min/1.73 m^2^; henceforth eGFR units are removed for ease of reading) between each eGFR equation. We compared baseline characteristics of participants included in this analysis across 4 categories of eGFR using ANOVA and *χ*^2^ test for continuous and categorical variables, respectively.

We then determined age-adjusted all-cause and CVD mortality rate (deaths/1000 person-years) during the follow-up time by category of baseline eGFR for each estimating equation. We calculated unadjusted hazard ratios for death by eGFR category using Cox proportional hazards regression. We further adjusted for factors determined a priori to be clinically relevant potential confounders of the association between renal function and death (age, race, BMI, DM, and HTN) and other factors that were associated with both predictor and outcomes. Thus, we present the following models: (1) an unadjusted model (site only); (2) a base model, further adjusted for age, race, and BMI; (3) a multivariate model, further adjusted for HTN and DM; and (4) a final model, further adjusted for CHD and self-reported health status.

Finally, we calculated a metric comparable to the net reclassification improvement (NRI) for each formula compared to the CKD-EPI_cr,cysc_ equation (see Appendix Table 2 for NRI formulae) [12]. The point estimate of the NRI is interpreted as follows: a positive NRI, total for the estimating equation evaluated means that the equation more often appropriately reclassifies to a HIGHER eGFR (better function) those participants who lived and/or to a LOWER eGFR (worse function) those who died when compared to the referent equation (CKD-EPI_cr,cysc_). We also present a category-free NRI which defines reclassification as 1 unit change in eGFR that allows us to provide estimates that may be more comparable across studies that may not choose the eGFR categories we have used in our analyses [13].

In secondary analyses, we repeated all of the above analyses expressing eGFR in quartiles as the outcome (see Appendix Tables 3 and 4). All significance levels reported were two-sided and all analyses were conducted using SAS 9.4 (SAS Institute Inc., Cary, NC).

## 3. Results

### 3.1. Prevalence by eGFR Categories and Baseline Characteristics

The mean age of women in this cohort was 79.5 ± 4.6 years and 90% were white. Mean BMI was 27 ± 5 kg/m^2^. Mean follow-up time was 9 ± 4 years. The BIS2 equation identified 47% and 10% as having eGFR <60 and eGFR <45, respectively, compared to 33% and 11% by the CKD-EPI_cr,cysc_ equation. The BIS1 equation identified 49% and 12% as having eGFR <60 and eGFR <45, respectively, compared to 24% and 8% for the CKD-EPI_cr_ equation (*p* < 0.001 for all pairwise comparisons, Table 1).

Women in a lower eGFR category, regardless of formula used, were older, more likely to be white, with poorer health status, and more likely to have a history of hypertension, stroke, CHD and CHF. In addition, women with lower CKD-EPI_cr,cysc_ and BIS2 eGFR had higher BMI. Worse renal function was associated with greater prevalence of DM, though this trend was statistically significant only across BIS2 eGFR category. Characteristics of participants according to category of CKD-EPI_cr,cysc_ are displayed in Table 2.

### 3.2. Estimated GFR and Risk of Death

Over the follow-up period, 667 (51.8%) women died overall and 239 (18.3%) of women experienced a CVD death. In unadjusted analyses, lower eGFR category (worse function) calculated using any one of the four equations was associated with a graded, increased risk of all-cause mortality that was similar in magnitude across eGFR formulae (Table 3). Women with eGFR <45 compared to those with eGFR ≥75 had a 2.9-fold higher risk of death by BIS1 eGFR and a 3.1-fold higher risk of death by CKD-EPI_cr_ eGFR (*p*-trend across category of eGFR <0.001 for both equations). Women with eGFR <45 compared to those with eGFR ≥75 had a 3.2-fold higher risk of death when eGFR was calculated using CKD-EPI_cr,cysc_ formula and a 3.7-fold higher risk of death when eGFR was estimated using BIS2 equation (*p*-trend across category of eGFR <0.001 for both equations). Further adjustment for age, race, BMI, comorbid conditions, and health status somewhat attenuated the point estimates of the associations, though point estimates of the association were similar between each BIS equation and its respective CKD-EPI comparator formula (HR [95% CI] for eGFR <45 versus ≥75: BIS1, 1.5 [1.1–2.0]; CKD-EPI_cr_, 1.7 [1.3–2.2]; BIS2, 2.0 [1.4–2.8]; CKD-EPI_cr,cysc_, 1.8 [1.4–2.3]). When we defined renal function by quartiles of eGFR, the point estimates were less robust, but similar significant trends across quartiles of eGFR were observed (Appendix Table 3).

When CVD mortality was substituted for all-cause mortality outcome in the analyses, lower eGFR category calculated using each one of the four equations was similarly associated with higher risk of CVD death across formulae (Table 4). Point estimates of the association of eGFR with CVD death in the final multivariable model were similar to those in the all-cause mortality analysis, despite borderline statistical significance. When renal function was expressed by quartiles of eGFR in analyses with CVD death as the outcome variable, we observed similar trends across quartiles, though the point estimates were less robust than when we used eGFR categories and were not statistically significant after adjusting for age, race, and BMI (Appendix Table 4).

Finally, in category-based NRI analyses (Table 5), the BIS1 equation when compared to the CKD-EPI_cr_ equation more appropriately categorized women who died to a lower eGFR category. However, to a greater degree, the BIS1 inappropriately categorized women who did not die to a lower eGFR category. Overall, the net reclassification favored less appropriate risk classification for BIS1 compared to that for the CKD-EPI_cr_, though this difference was not statistically significant. We observed similar results for the BIS2 compared to the CKD-EPI_cr,cysc_. Findings were not altered when analyses were repeated using the category-free classification of eGFR. When CVD mortality was the outcome, we noted similar risk-reclassification patterns in both category-based and category-free NRI analyses, though the point estimate of NRI for nonevents was more strongly in the direction of inappropriate recategorization to a lower eGFR for those who did not experience a CVD death. None of the NRI values were statistically different from those of the referent CKD-EPI equation.

## 4. Discussion

In this cohort of community-dwelling older women, lower eGFR irrespective of estimating equation used was associated with a higher risk of all-cause mortality and CVD death. The BIS eGFR equations, which were developed specifically to estimate kidney function in older adults, did not outperform the KDIGO-recommended CKD-EPI equations to predict death including CVD mortality in this population. Thus, our findings lend no support to the replacement of the CKD-EPI equations with the BIS equations to predict death in older women.

We found that nearly half of our participants had an eGFR <60 by either BIS equation compared to 27% and 33% for the CKD-EPI_cr_ and CKD-EPI_cr,cysc_ equations. These results are consistent with those of other studies examining the prevalence of CKD by the BIS and CKD-EPI formulae in both older women and men [9, 14–16]. The prevalence of eGFR by either BIS equation was similar to that of directly measured GFR in other studies of older adults similar in age to our cohort, though these studies were not limited to women [9, 16]. In addition, in previous studies of older adults, the BIS equations consistently identified a higher prevalence of CKD compared to the CKD-EPI equations [14, 15, 17]. For example, in an external validation study of the BIS equations in 394 men and women of median age 80 years, Alshaer et al. found that the BIS equations identified a greater proportion of the population as having lower eGFR, while the CKD-EPI equations overestimated renal function when compared to measured GFR [17]. Taken together, our findings in concert with those of others confirm that the BIS equations tend to identify a higher proportion of older adults as having CKD.

Furthermore, findings from other studies of older men and women have been consistent with our observation that the BIS equations do not outperform the CKD-EPI equations in predicting all-cause and CVD mortality [14, 15, 18, 19]. A population-based prospective study of Italian men and women over 85 years old found that after 5 years' follow-up time the BIS1 did not outperform other equations including the CKD-EPI_cr_ equation to predict mortality. Another population-based cohort study of 1017 German men and women (59% female) aged 71–75 years observed that the BIS2 equation more inappropriately classified participants who did not die to a lower eGFR category when compared to the CKD-EPI_cr,cysc_ [18]. Finally, a study of community-dwelling older US men similarly found that the BIS2 equation compared to the CKD-EPI_cr,cysc_ formula more inappropriately reclassified men to lower eGFR who did not die. While none of these studies were performed solely in women, their findings corroborate our observation that the BIS equations do not outperform the CKD-EPI equations with respect to mortality risk prediction.

The clinical implication of our study is of importance to both clinicians and researchers. The 2012 KDIGO guideline recommends a paradigm shift from the clinical standard MDRD eGFR equation to the CKD-EPI equations to estimate GFR [7]. For populations in which SCr-based estimates may be less reliable (e.g., older age), Scysc-based CKD-EPI equations are recommended [7]. However, the guidelines note that should other novel equations arise that are more applicable to certain populations, consideration of use of those equations is reasonable [7]. Considering that several novel equations have been developed in the past 10 years, this leaves the practitioner and researcher in a quandary regarding the best equation to meet their needs. To that end, our study provides useful information regarding the performance of a new equation specifically developed for community-dwelling older adults compared with that of guideline-recommended equations. Based upon our findings, the BIS equations should not replace the CKD-EPI equations to predict death on older community-dwelling women.

Our study has several key strengths that set it apart from other studies. First, to our knowledge, we are the first study to test the performance of the BIS equations against the CKD-EPI equations to predict death in a cohort entirely composed of older women. Our cohort had long-term follow-up and accurate ascertainment of outcomes and covariate data. Furthermore, we present equations based not only on SCr but also on SCr and Scysc, both of which are traceable to the international reference standard. This allowed us to compare the predictive validity of each BIS equation against a comparable CKD-EPI equation. In addition, the incorporation of Scysc in older adults may be important given studies that have shown that Scysc-based estimates are more sensitive predictors of death in older adults [20].

We also note that our study has limitations to consider. We lack generalizability to male populations given our cohort is entirely female. However, we have previously published similar findings in a cohort consisting only of older men, thus corroborating that our findings are consistent in women and men. In addition, our cohort is largely Caucasian and though 10% are Black, there is limited racial or ethnic diversity otherwise. The BIS equations were developed in entirely Caucasian population which makes SOF a fitting cohort in which to test the BIS equations. However, because of the limited diversity in the BIS Study, there is no race adjustment for those equations which may limit their reliability among Black SOF participants. Also, we lack information regarding a complementary marker of renal function and predictor of CKD-related death, albuminuria. However, because the purpose of this analysis was purely to compare the performance of 4 eGFR formulae to predict death, the addition of albuminuria across equations would not likely have changed our observations. In addition, we lack information on the cause of CKD, if present, or episodes of acute kidney injury during the follow-up period. These factors may be related to risk of CKD-related outcomes such as mortality. However, we have adjusted for common comorbid conditions that cause CKD such as DM and HTN. Finally, we cannot be certain that our estimates of renal function represent the true baseline eGFR of the participants because we do not have prior estimates of renal function in these population-based cohorts in which we measured SCr from stored serum from a single time point.

To sum, the BIS formulae identify a higher proportion of older community-dwelling women as having CKD. Our results suggest that the BIS equations do not outperform guideline-recommended CKD-EPI equations. Thus, with respect to predicting risk of death from estimated renal function in older women, our findings do not lend support for incorporation of the BIS equations into clinical practice or research.

## Supplementary Material

The supplementary materials provided include four tables. The first shows the 4 equations used to estimate GFR in this manuscript. The second table outlines the net reclassification improvement formulae used. Finally, the last two tables display the results of Cox proportional hazards regression analysis using quartiles of eGFR instead of categories of eGFR as the predictor of all-cause or cardiovascular death.

## Figures and Tables

**Table 1:** Prevalence of eGFR category using 4 different estimating equations.

eGFR category, mL/min/1.73 m^2^	GFR estimating equation, *n* (%)			
	BIS1	CKD-EPI_cr_	BIS2	CKD-EPI_cr,cysc_
≥75	159 (12.3)	622 (48.3)	182 (14.1)	432 (33.5)
60 to <75	495 (38.4)	358 (27.8)	498 (38.6)	430 (33.4)
45 to <60	481 (37.3)	209 (16.2)	485 (37.6)	284 (22.0)
<45	154 (12.0)	100 (7.8)	124 (9.6)	143 (11.1)

eGFR, estimated glomerular filtration rate; BIS, Berlin Initiative Study; CKD-EPI, Chronic Kidney Disease Epidemiology Collaboration; cr, creatinine; cysc, cystatin C.

**Table 2:** Clinical characteristics of SOF participants by CKD-EPI_cr,cysc_ eGFR category.

Baseline characteristics	Overall (*N* = 1289)	CKD-EPI_cr,cysc_ eGFR category (mL/min/1.73 m^2^)				*p* value
		≥75	60 to 74	45–59	<45	
		(*N* = 432)	(*N* = 430)	(*N* = 284)	(*N* = 143)	
Age, years, mean (SD)	79.5 (4.6)	77.7 (4.1)	79.4 (4.2)	81.3 (4.8)	82.0 (4.8)	<0.001
Race/ethnicity, *n* (%)						<0.001
White	1159 (89.9)	355 (82.2)	399 (92.8)	270 (95.1)	135 (94.4)	
Black	130 (10.1)	77 (17.8)	31 (7.2)	14 (4.9)	8 (5.6)	
Education, *n* (%)						0.031
Less than high school	229 (17.8)	62 (14.4)	69 (16.1)	65 (23.0)	33 (23.1)	
High school	538 (41.8)	190 (44.1)	182 (42.4)	116 (41.0)	50 (35.0)	
College/grad school	519 (40.4)	179 (41.5)	178 (41.5)	102 (36.0)	60 (42.0)	
Body mass index, kg/m^2^, mean (SD)	26.8 (5.0)	26.1 (4.9)	27.1 (5.0)	27.0 (4.8)	27.4 (5.2)	0.008
Current smoker, *n* (%)	58 (4.5)	27 (6.3)	15 (3.5)	12 (4.2)	4 (2.8)	0.167
Self-rated health good/excellent, *n* (%)	1035 (80.4)	366 (84.7)	357 (83.0)	217 (76.4)	95 (66.9)	<0.001
Hypertension^*∗*^, *n* (%)	795 (61.7)	235 (54.4)	242 (56.3)	196 (69.0)	122 (85.3)	<0.001
Diabetes mellitus^*∗*^, *n* (%)	95 (7.4)	33 (7.6)	23 (5.4)	23 (8.1)	16 (11.3)	0.112
Self-reported stroke, *n* (%)	60 (4.7)	18 (4.2)	10 (2.3)	20 (7.0)	12 (8.5)	0.003
Self-reported coronary heart disease^†^, *n* (%)	246 (19.1)	73 (16.9)	67 (15.6)	62 (21.8)	44 (31.0)	<0.001
Self-reported congestive heart failure, *n* (%)	65 (5.1)	21 (4.9)	12 (2.8)	20 (7.0)	12 (8.5)	0.016
CKD-EPI_cr,cysc_ eGFR, mean (SD)	66.7 (16.7)	84.6 (7.1)	67.6 (4.2)	53.5 (4.1)	36.3 (7.2)	<0.001
Follow-up time, years, mean (SD)	9.2 (4.0)	10.2 (3.8)	9.6 (3.9)	8.3 (4.0)	6.8 (4.1)	<0.001

eGFR, estimated glomerular filtration rate; CKD-EPI, Chronic Kidney Disease Epidemiology Collaboration; cr, creatinine; cysc, cystatin C.

^*∗*^As determined by self-report or any disease-specific medication use.

^†^Coronary heart disease includes myocardial infarction, angina, and other heart diseases.

**Table 3:** Association of eGFR category and all-cause mortality.

Estimating equation category, mL/min/1.73 m^2^	Age adjusted incidence rate per 1,000 person years (95% CI)	Hazard ratio (95% confidence interval)			
		Unadjusted (site only)	Base model^*∗*^	MV model^†^	Final model^‡^
BIS1					
≥75	75.7 (29.7, 121.6)	1.0 (reference)	1.0 (reference)	1.0 (reference)	1.0 (reference)
60 to <75	49.6 (42.0, 57.2)	0.9 (0.7, 1.2)	0.8 (0.6, 1.1)	0.9 (0.7, 1.1)	0.9 (0.7, 1.1)
45 to <60	55.7 (48.5, 62.8)	1.4 (1.1, 1.8)	1.0 (0.8, 1.4)	1.0 (0.8, 1.4)	1.0 (0.8, 1.3)
<45	90.3 (67.2, 113.5)	2.9 (2.2, 3.9)	1.7 (1.2, 2.3)	1.6 (1.1, 2.1)	1.5 (1.1, 2.0)
*p*-trend		<0.001	<0.001	0.002	0.006
CKD-EPI_cr_					
≥75	52.3 (45.7, 58.9)	1.0 (reference)	1.0 (reference)	1.0 (reference)	1.0 (reference)
60 to <75	56.5 (48.2, 64.9)	1.4 (1.2, 1.7)	1.2 (1.0, 1.4)	1.1 (0.9, 1.4)	1.1 (0.9, 1.3)
45 to <60	57.5 (45.6, 69.4)	1.6 (1.3, 2.0)	1.2 (1.0, 1.5)	1.1 (0.9, 1.4)	1.1 (0.9, 1.4)
<45	91.5 (63.1, 119.9)	3.1 (2.4, 4.0)	2.0 (1.5, 2.7)	1.8 (1.4, 2.4)	1.7 (1.3, 2.2)
*p*-trend		<0.001	<0.001	<0.001	0.003
BIS2					
≥75	43.6 (29.6, 57.6)	1.0 (reference)	1.0 (reference)	1.0 (reference)	1.0 (reference)
60 to <75	49.3 (42.1, 56.4)	1.2 (0.9, 1.6)	1.1 (0.8, 1.4)	1.1 (0.8, 1.5)	1.1 (0.8, 1.5)
45 to <60	56.8 (49.8, 63.9)	1.9 (1.4, 2.5)	1.4 (1.0, 1.9)	1.4 (1.0, 1.9)	1.4 (1.0, 1.9)
<45	92.1 (72.0, 112.1)	3.7 (2.6, 5.1)	2.3 (1.6, 3.3)	2.1 (1.5, 3.0)	2.0 (1.4, 2.8)
*p*-trend		<0.001	<0.001	<0.001	<0.001
CKD-EPI_cr,cysc_					
≥75	49.7 (41.6, 57.8)	1.0 (reference)	1.0 (reference)	1.0 (reference)	1.0 (reference)
60 to <75	53.3 (45.7, 60.8)	1.3 (1.1, 1.6)	1.1 (0.9, 1.3)	1.1 (0.9, 1.3)	1.1 (0.9, 1.3)
45 to <60	61.8 (50.9, 72.6)	2.0 (1.6, 2.4)	1.4 (1.1, 1.8)	1.3 (1.1, 1.7)	1.3 (1.0, 1.6)
<45	101.7 (69.8, 133.5)	3.2 (2.5, 4.0)	2.1 (1.6, 2.8)	1.9 (1.5, 2.5)	1.8 (1.4, 2.3)
*p*-trend		<0.001	<0.001	<0.001	<0.001

eGFR, estimated glomerular filtration rate; BIS, Berlin Initiative Study; CKD-EPI, Chronic Kidney Disease Epidemiology Collaboration; cr, creatinine; cysc, cystatin C.

^*∗*^Adjusted for age, race, and body mass index.

^†^Adjusted for age, race, body mass index, hypertension, and diabetes mellitus.

^‡^Adjusted for age, race, body mass index, hypertension, diabetes mellitus, history of CHD, and self-reported health status.

**Table 4:** Association of eGFR category and cardiovascular mortality.

Estimating equation category, mL/min/1.73 m^2^	Age adjusted incidence rate per 1,000 person years (95% CI)	Hazard ratio (95% confidence interval)			
		Unadjusted (site only)	Base model^*∗*^	MV model^†^	Final model^‡^
BIS1					
≥75	45.7 (0.8, 90.6)	1.0 (reference)	1.0 (reference)	1.0 (reference)	1.0 (reference)
60 to <75	16.5 (11.7, 21.3)	0.8 (0.5, 1.2)	0.7 (0.4, 1.1)	0.8 (0.5, 1.2)	0.8 (0.5, 1.2)
45 to <60	18.7 (14.6, 22.8)	1.3 (0.8, 2.0)	0.9 (0.6, 1.3)	0.9 (0.6, 1.4)	0.9 (0.6, 1.3)
<45	38.4 (22.9, 53.9)	3.4 (2.2, 5.5)	1.8 (1.1, 2.9)	1.6 (0.9, 2.6)	1.4 (0.9, 2.4)
*p*-trend		<0.001	0.005	0.036	0.099
CKD-EPI_cr_					
≥75	19.1 (14.7, 23.4)	1.0 (reference)	1.0 (reference)	1.0 (reference)	1.0 (reference)
60 to <75	19.1 (14.4, 23.8)	1.4 (1.1, 2.0)	1.1 (0.8, 1.6)	1.1 (0.8, 1.5)	1.0 (0.7, 1.4)
45 to <60	20.3 (13.0, 27.6)	1.6 (1.1, 2.4)	1.2 (0.8, 1.7)	1.0 (0.7, 1.5)	1.0 (0.7, 1.5)
<45	39.7 (21.9, 57.5)	4.1 (2.7, 6.1)	2.6 (1.7, 4.0)	2.1 (1.3, 3.2)	1.8 (1.2, 2.8)
*p*-trend		<0.001	<0.001	0.016	0.043
BIS2					
≥75	19.4 (9.6, 29.2)	1.0 (reference)	1.0 (reference)	1.0 (reference)	1.0 (reference)
60 to <75	16.4 (12.1, 20.8)	0.9 (0.5, 1.5)	0.8 (0.5, 1.4)	0.9 (0.5, 1.5)	0.8 (0.5, 1.4)
45 to <60	19.8 (15.6, 23.9)	1.6 (0.9, 2.6)	1.1 (0.7, 1.8)	1.1 (0.7, 1.9)	1.1 (0.6, 1.8)
<45	37.5 (25.5, 49.6)	3.7 (2.2, 6.3)	2.2 (1.3, 3.8)	1.9 (1.1, 3.4)	1.7 (0.9, 3.0)
*p*-trend		<0.001	<0.001	0.001	0.006
CKD-EPI_cr,cysc_					
≥75	17.5 (12.4, 22.6)	1.0 (reference)	1.0 (reference)	1.0 (reference)	1.0 (reference)
60 to <75	17.8 (13.6, 21.9)	1.3 (0.9, 1.9)	1.1 (0.8, 1.6)	1.1 (0.8, 1.6)	1.1 (0.8, 1.6)
45 to <60	21.5 (15.8, 27.3)	2.2 (1.5, 3.2)	1.5 (1.0, 2.2)	1.4 (0.9, 2.0)	1.3 (0.9, 1.9)
<45	48.3 (21.4, 75.2)	3.9 (2.6, 5.9)	2.5 (1.7, 3.8)	2.0 (1.3, 3.1)	1.8 (1.2, 2.8)
*p*-trend		<0.001	<0.001	0.002	0.011

eGFR, estimated glomerular filtration rate; BIS, Berlin Initiative Study; CKD-EPI, Chronic Kidney Disease Epidemiology Collaboration; cr, creatinine; cysc, cystatin C.

^*∗*^Adjusted for age, race, and body mass index.

^†^Adjusted for age, race, body mass index, hypertension, and diabetes mellitus.

^‡^Adjusted for age, race, body mass index, hypertension, diabetes mellitus, history of CHD, and self-reported health status.

**Table 5:** Net reclassification improvement (NRI)^†^ for GFR estimating equations.

Estimating equation by outcome	Category-based^*∗*^		Category-free^‡^	
	NRI (95% CI)	*p* value	NRI (95% CI)	*p*-value
*All-cause mortality*				
BIS1 (compared to reference CKD-EPI_cr_)				
Events	0.63 (0.60, 0.67)	<0.001	0.91 (0.88, 0.94)	<0.001
Nonevents	−0.64 (−0.68, −0.61)	<0.001	−0.96 (−0.98, −0.95)	<0.001
Total NRI	−0.01 (−0.06, 0.04)	0.815	−0.05 (−0.09, −0.02)	0.336
BIS2 (compared to reference CKD-EPI_cr,cysc_)				
Events	0.46 (0.42, 0.50)	<0.001	0.85 (0.82, 0.89)	<0.001
Nonevents	−0.47 (−0.51, −0.44)	<0.001	−0.94 (−0.96, −0.92)	<0.001
Total NRI	−0.02 (−0.07, 0.04)	0.655	−0.09 (−0.13, −0.05)	0.108
*Cardiovascular mortality*				
BIS1 (compared to reference CKD-EPI_cr_)				
Events	0.62 (0.56, 0.68)	<0.001	0.89 (0.84, 0.95)	<0.001
Nonevents	−0.64 (−0.67, −0.61)	<0.001	−0.95 (−0.96, −0.93)	<0.001
Total NRI	−0.02 (−0.09, 0.05)	0.682	−0.05 (−0.11, 0.004)	0.459
BIS2 (compared to reference CKD-EPI_cr,cysc_)				
Events	0.43 (0.36, 0.50)	<0.001	0.80 (0.74, 0.87)	<0.001
Nonevents	−0.47 (−0.50, −0.44)	<0.001	−0.91 (−0.94, −0.89)	<0.001
Total NRI	−0.04 (−0.11, 0.03)	0.394	−0.11 (−0.18, −0.05)	0.097

GFR, glomerular filtration rate; NRI, net reclassification improvement; BIS, Berlin Initiative Study; CKD-EPI, Chronic Kidney Disease Epidemiology Collaboration; cr, creatinine; cysc, cystatin C.

^*∗*^Based on categorization ≥75, 60 to <75, 45 to <60, <45 mL/min/1.73 m^2^.

^†^NRI, total = NRI, events + NRI, nonevents

NRI, events = [proportion of participants reclassified downward to a lower eGFR category for people who died MINUS proportion of participants reclassified upward for people who died]

NRI, nonevents = [proportion of participants reclassified upward to a higher eGFR category for people who did not die MINUS the proportion of participants reclassified downward for people who did not die].

^‡^Based upon a 1 mL/min/1.73 m^2^ change in estimated glomerular filtration rate.

## References

[1] O'Hare A. M., Choi A. I., Bertenthal D. (2007). Age affects outcomes in chronic kidney disease. *Journal of the American Society of Nephrology*.

[2] Foley R. N., Murray A. M., Li S. (2005). Chronic kidney disease and the risk for cardiovascular disease, renal replacement, and death in the United States medicare population, 1998 to 1999. *Journal of the American Society of Nephrology*.

[3] Keith D. S., Nichols G. A., Gullion C. M., Brown J. B., Smith D. H. (2004). Longitudinal follow-up and outcomes among a population with chronic kidney disease in a large managed care organization. *Archives of Internal Medicine*.

[4] Levey A. S., Inker L. A., Coresh J. (2015). Chronic kidney disease in older people. *Journal of the American Medical Association*.

[5] Glassock R., Delanaye P., El Nahas M. (2015). An age-calibrated classification of chronic kidney disease. *JAMA - Journal of the American Medical Association*.

[6] Inker L. A., Schmid C. H., Tighiouart H. (2012). Estimating glomerular filtration rate from serum creatinine and cystatin C. *New England Journal of Medicine*.

[7] Kidney Disease: Improving Global Outcomes (KDIGO) (2013). KDIGO 2012 Clinical Practice Guideline for the evaluation and management of chronic kidney disease. *Kidney International Supplement*.

[8] Levey A. S., Stevens L. A., Schmid C. H. (2009). A new equation to estimate glomerular filtration rate. *Annals of Internal Medicine*.

[9] Schaeffner E. S., Ebert N., Delanaye P. (2012). Two novel equations to estimate kidney function in persons aged 70 years or older. *Annals of Internal Medicine*.

[10] Cummings S. R., Nevitt M. C., Browner W. S. (1995). Risk factors for hip fracture in white women. *New England Journal of Medicine*.

[11] Vogt M. T., Rubin D. A., Palermo L. (2003). Lumbar spine listhesis in older African American women. *Spine Journal*.

[12] Pencina M. J., D'Agostino S., D'Agostino J., Vasan R. S. (2008). Evaluating the added predictive ability of a new marker: from area under the ROC curve to reclassification and beyond. *Statistics in Medicine*.

[13] Pencina M. J., D'Agostino S., Steyerberg E. W. (2011). Extensions of net reclassification improvement calculations to measure usefulness of new biomarkers. *Statistics in Medicine*.

[14] Van Pottelbergh G., Vaes B., Adriaensen W. (2014). The glomerular filtration rate estimated by new and old equations as a predictor of important outcomes in elderly patients. *BMC Medicine*.

[15] Mandelli S., Riva E., Tettamanti M., Detoma P., Giacomin A., Lucca U. (2015). Mortality prediction in the oldest old with five different equations to estimate glomerular filtration rate: The Health and Anemia population-based study. *PLoS ONE*.

[16] Fan L., Levey A. S., Gudnason V. (2015). Comparing GFR estimating equations using cystatin C and creatinine in elderly individuals. *Journal of the American Society of Nephrology*.

[17] Alshaer I. M., Kilbride H. S., Stevens P. E. (2014). External validation of the berlin equations for estimation of GFR in the elderly. *American Journal of Kidney Diseases*.

[18] Emrich I. E., Pickering J. W., Schöttker B. (2015). Comparison of the performance of 2 GFR estimating equations using creatinine and cystatin C to predict adverse outcomes in elderly individuals. *American Journal of Kidney Diseases*.

[19] Canales M. T., Blackwell T., Ishani A. (2016). Estimated GFR and mortality in older men: are all eGFR formulae equal?. *American Journal of Nephrology*.

[20] Shlipak M. G., Matsushita K., Arnlov J. (2013). Cystatin C versus creatinine in determining risk based on kidney function. *New England Journal of Medicine*.

