# Supplementary material for: Renal Function and Death in Older Women: Which eGFR Formula Should We Use?
Source: Int J Nephrol. 2017 Mar 29;2017:8216878. doi: 10.1155/2017/8216878 (PMC5390547; doi:10.1155/2017/8216878)
Supplement: Supplementary file 1 — The supplementary materials provided include four tables. The first shows the 4 equations used to estimate GFR in this manuscript. The second table outlines the net reclassification improvement formulae used. Finally, the last two tables display the results of Cox proportional hazards regression analysis using quartiles of eGFR instead of categories of eGFR as the predictor of all-cause or cardiovascular death. [file 8216878.f1.docx]

**Appendix Table 1. eGFR Formulae Equations**

| Estimating Equation Category | Formula |
| --- | --- |
| CKD-EPI_cr_ (6) | SCr ≤0.9: 141 × (Scr/0.9)^−0.411^ × 0.993^Age^ [× 1.159 if black] |
|  | SCr >0.9: 141 × (Scr/0.9)^−1.209^ × 0.993^Age^ [× 1.159 if black] |
| BIS1 (8) | 3736 × creatinine^−0.87^ × age^−0.95^ × 0.82 |
| CKD-EPI_cr,cysc_ (6) | SCr ≤0.9 and SCysc ≤0.8: 135 × (SCr/0.9)^−0.207^ × (SCysc/0.8)^−0.375^ × 0.995^Age^ [× 1.08 if black] |
|  | SCr ≤0.9 and SCysc >0.8: 135 × (SCr/0.9)^−0.207^ × (SCysc/0.8^)−0.711^ × 0.995^Age^ [× 1.08 if black] |
|  | SCr >0.9 and SCysc ≤0.8: 135 × (SCr/0.9)^−0.601^ × (SCysc/0.8)^−0.375^ × 0.995^Age^ [× 1.08 if black] |
|  | SCr >0.9 and SCysc >0.8: 135 × (SCr/0.9)^−0.601^ × (SCysc/0.8)^−0.711^ × 0.995^Age^ [× 1.08 if black] |
| BIS2 (8) | 767 × SCysc^−0.61^ × SCr^−0.40^ × age^−0.57^ × 0.87 |

Abbreviations: SCr, serum creatinine; SCysc, serum cystatin C

**Appendix Table 2. Net Reclassification Improvement (NRI) Formulae**

|  | Definition |
| --- | --- |
| NRI, events | proportion of participants reclassified downward to a lower eGFR category for people who died MINUS proportion of participants reclassified upward for people who died |
| NRI, nonevents | proportion of participants reclassified upward to a higher eGFR category for people who did not die MINUS the proportion of participants reclassified downward for people who did not die |
| NRI, total | NRI, events + NRI, nonevents |

**Appendix Table 3. Association of eGFR Quartile and All-Cause Mortality**

|  |  | Relative Hazard (95% Confidence Interval) | | | |
| --- | --- | --- | --- | --- | --- |
| Estimating Equation Category, mL/min/1.73m^2^ | Age Adjusted Incidence Rate per 1,000 Person Years (95% CI) | Unadjusted  (site only) | Base Model* | MV Model† | Final Model‡ |
| BIS1 |  |  |  |  |  |
| Quartile 4 | 61.6 (43.7, 79.5) | 1.0 (reference) | 1.0 (reference) | 1.0 (reference) | 1.0 (reference) |
| Quartile 3 | 52.3 (38.4, 66.3) | 1.1 (0.8, 1.3) | 0.9 (0.7, 1.1) | 0.9 (0.7, 1.1) | 0.9 (0.7, 1.1) |
| Quartile 2 | 57.0 (48.1, 65.8) | 1.5 (1.2, 1.9) | 1.1 (0.9, 1.4) | 1.1 (0.9, 1.4) | 1.1 (0.8, 1.3) |
| Quartile 1 | 66.9 (55.7, 78.2) | 2.2 (1.8, 2.7) | 1.4 (1.1, 1.7) | 1.3 (1.0, 1.6) | 1.3 (0.9, 1.6) |
| p-trend |  | <0.001 | 0.002 | 0.014 | 0.026 |
| CKD-EPI_cr_ |  |  |  |  |  |
| Quartile 4 | 53.6 (40.4, 66.7) | 1.0 (reference) | 1.0 (reference) | 1.0 (reference) | 1.0 (reference) |
| Quartile 3 | 48.7 (40.7, 56.7) | 1.3 (1.0, 1.6) | 0.9 (0.7, 1.1) | 0.9 (0.7, 1.2) | 0.9 (0.7, 1.2) |
| Quartile 2 | 58.7 (49.7, 67.8) | 1.7 (1.3, 2.1) | 1.2 (0.9, 1.5) | 1.2 (0.9, 1.5) | 1.1 (0.9, 1.4) |
| Quartile 1 | 65.7 (54.8, 76.6) | 2.2 (1.8, 2.8) | 1.3 (1.0, 1.7) | 1.2 (0.9, 1.6) | 1.2 (0.9, 1.5) |
| p-trend |  | <0.001 | 0.002 | 0.015 | 0.027 |
| BIS2 |  |  |  |  |  |
| Quartile 4 | 45.1 (35.2, 55.1) | 1.0 (reference) | 1.0 (reference) | 1.0 (reference) | 1.0 (reference) |
| Quartile 3 | 51.0 (42.6, 59.5) | 1.3 (1.0, 1.6) | 1.1 (0.9, 1.4) | 1.1 (0.9, 1.4) | 1.1 (0.8, 1.4) |
| Quartile 2 | 56.3 (47.5, 65.1) | 1.7 (1.4, 2.4) | 1.3 (1.1, 1.7) | 1.3 (1.0, 1.7) | 1.3 (1.0, 1.7) |
| Quartile 1 | 78.2 (62.6, 93.8) | 2.8 (2.2, 3.4) | 1.8 (1.4, 2.3) | 1.7 (1.3, 2.1) | 1.6 (1.2, 2.0) |
| p-trend |  | <0.001 | <0.001 | <0.001 | <0.001 |
| CKD-EPI_cr,cys_ |  |  |  |  |  |
| Quartile 4 | 44.2 (35.1, 53.3) | 1.0 (reference) | 1.0 (reference) | 1.0 (reference) | 1.0 (reference) |
| Quartile 3 | 51.0 (42.9, 59.1) | 1.3 (1.2, 1.7) | 1.1 (0.9, 1.4) | 1.1 (0.8, 1.4) | 1.0 (0.8, 1.3) |
| Quartile 2 | 62.7 (48.5, 76.9) | 1.8 (1.4, 2.2) | 1.3 (1.1, 1.7) | 1.3 (1.0, 1.7) | 1.3 (1.0, 1.7) |
| Quartile 1 | 76.3 (61.1, 91.6) | 2.7 (2.2, 3.4) | 1.8 (1.4, 2.2) | 1.6 (1.2, 2.0) | 1.5 (1.2, 1.9) |
| p-trend |  | <0.001 | <0.001 | <0.001 | <0.001 |

Abbreviations: eGFR, estimated glomerular filtration rate; BIS, Berlin Initiative Study; CKD-EPI, Chronic Kidney Disease Epidemiology Collaboration; cr, creatinine; cysc, cystatin c

Quartile ranges: Q1: 13.489-50.200; Q2: 50.289-59.167; Q3: 59.176-67.550; Q4: ≥67.551 mL/min/1.73m^2^

*adjusted for age, race, and body mass index

†adjusted for age, race, body mass index, hypertension, diabetes mellitus

‡adjusted for age, race, body mass index, hypertension, diabetes mellitus, history of CHD and self-reported health status

**Appendix Table 4.** **Association of eGFR Quartile and Cardiovascular Mortality**

|  |  | Relative Hazard (95% Confidence Interval) | | | |
| --- | --- | --- | --- | --- | --- |
| Estimating Equation Category, mL/min/1.73m^2^ | Age Adjusted Incidence Rate per 1,000 Person Years (95% CI) | Unadjusted  (site only) | Base Model* | MV Model† | Final Model‡ |
| BIS1 |  |  |  |  |  |
| Quartile 4 | 29.2 (12.8, 45.6) | 1.0 (reference) | 1.0 (reference) | 1.0 (reference) | 1.0 (reference) |
| Quartile 3 | 15.2 (10.2, 20.2) | 1.0 (0.6, 1.5) | 0.8 (0.5, 1.2) | 0.8 (0.6, 1.3) | 0.8 (0.6, 1.3) |
| Quartile 2 | 20.4 (15.1, 25.6) | 1.6 (1.1, 2.3) | 1.1 (0.7, 1.6) | 1.0 (0.7, 1.5) | 1.0 (0.7, 1.4) |
| Quartile 1 | 25.7 (18.5, 32.9) | 2.4 (1.7, 3.5) | 1.4 (0.9, 2.1) | 1.2 (0.8, 1.8) | 1.2 (0.8, 1.8) |
| p-trend |  | <0.001 | 0.039 | 0.190 | 0.304 |
| CKD-EPI_cr_ |  |  |  |  |  |
| Quartile 4 | 25.8 (14.5, 37.1) | 1.0 (reference) | 1.0 (reference) | 1.0 (reference) | 1.0 (reference) |
| Quartile 3 | 16.1 (11.2 20.9) | 1.0 (0.7, 1.5) | 0.7 (0.5, 1.0) | 0.7 (0.5, 1.1) | 0.7 (0.5, 1.1) |
| Quartile 2 | 19.6 (14.5, 24.7) | 1.5 (1.0, 2.2) | 1.0 (0.7, 1.5) | 1.0 (0.7, 1.4) | 0.9 (0.6, 1.4) |
| Quartile 1 | 25.2 (18.2, 32.1) | 2.2 (1.6, 3.2) | 1.2 (0.8, 1.8) | 1.1 (0.7, 1.6) | 1.0 (0.7, 1.6) |
| p-trend |  | <0.001 | 0.053 | 0.250 | 0.383 |
| BIS2 |  |  |  |  |  |
| Quartile 4 | 17.1 (9.6, 24.7) | 1.0 (reference) | 1.0 (reference) | 1.0 (reference) | 1.0 (reference) |
| Quartile 3 | 18.3 (13.1, 23.4) | 1.4 (0.9, 2.1) | 1.2 (0.8, 1.8) | 1.2 (0.8, 1.8) | 1.1 (0.8, 1.8) |
| Quartile 2 | 18.7 (13.8, 23.6) | 1.9 (1.3, 2.8) | 1.3 (0.9, 2.0) | 1.3 (0.9, 2.0) | 1.3 (0.8, 2.0) |
| Quartile 1 | 29.0 (21.3, 36.7) | 3.5 (2.3, 5.1) | 2.1 (1.4, 3.2) | 1.8 (1.2, 2.7) | 1.6 (1.1, 2.5) |
| p-trend |  | <0.001 | <0.001 | 0.005 | 0.021 |
| CKD-EPI_cr,cys_ |  |  |  |  |  |
| Quartile 4 | 16.9 (10.6, 23.1) | 1.0 (reference) | 1.0 (reference) | 1.0 (reference) | 1.0 (reference) |
| Quartile 3 | 17.6 (12.6, 22.5) | 1.3 (0.9, 1.9) | 1.0 (0.7, 1.6) | 1.0 (0.7, 1.6) | 1.0 (0.6, 1.5) |
| Quartile 2 | 18.7 (13.8, 23.6) | 1.7 (1.1, 2.5) | 1.2 (0.8, 1.9) | 1.2 (0.8, 1.8) | 1.2 (0.8, 1.8) |
| Quartile 1 | 28.5 (21.0, 36.0) | 3.1 (2.1, 4.5) | 1.9 (1.3, 2.9) | 1.6 (1.1, 2.4) | 1.4 (0.9, 2.2) |
| p-trend |  | <0.001 | <0.001 | 0.012 | 0.047 |

Abbreviations: eGFR, estimated glomerular filtration rate; BIS, Berlin Initiative Study; CKD-EPI, Chronic Kidney Disease Epidemiology Collaboration; cr, creatinine; cysc, cystatin c

Quartile ranges: Q1: 13.489-50.200; Q2: 50.289-59.167; Q3: 59.176-67.550; Q4: ≥67.551 mL/min/1.73m^2^

*adjusted for age, race, and body mass index

†adjusted for age, race, body mass index, hypertension, diabetes mellitus

‡adjusted for age, race, body mass index, hypertension, diabetes mellitus, history of CHD and self-reported health status
